# Risk of coronary events 55 Years after Thymic irradiation in the Hempelmann cohort

**DOI:** 10.1186/s40959-018-0027-0

**Published:** 2018-02-17

**Authors:** Michael Jacob Adams, Susan G. Fisher, Steven E. Lipshultz, Roy E. Shore, Louis S. Constine, Marilyn Stovall, Ann Dozier, Kelly Thevenet-Morrison, Robert Block, Ronald G. Schwartz, Thomas A. Pearson

**Affiliations:** 1 Department of Public Health Sciences, University of Rochester School of Medicine and Dentistry, Rochester, New York USA; 2 Department of Clinical Sciences, Lewis Katz School of Medicine Temple University, Philadelphia, PA USA; 3 Department of Pediatrics, Wayne State University School of Medicine, Children’ s Hospital of Michigan, and the Karmanos Cancer Institute, Detroit, MI USA; 4 Department of Environmental Medicine, New York University School of Medicine, New York, NY USA; 5 Departments of Radiation Oncology and Pediatrics, University of Rochester School of Medicine and Dentistry and the James P. Wilmot Cancer Center, Rochester, New York USA; 6 Department of Radiation Physics, University of Texas, M.D. Anderson Cancer Center, Houston, Texas USA; 7 Cardiology Division, Department of Medicine, University of Rochester School of Medicine and Dentistry, Rochester, New York USA; 8 Nuclear Medicine Division, Department of Imaging Sciences, University of Rochester School of Medicine and Dentistry, Rochester, New York USA; 9 Department of Epidemiology, Schools of Public Health and Health Professions and of Medicine, University of Florida, Gainesville, Florida USA; 10 Department of Community and Preventive Medicine, University of Rochester School of Medicine and Dentistry, 601 Elmwood Avenue, Box 644, Rochester, New York USA

**Keywords:** Cardiovascular disease, Coronary heart disease, Childhood cancer, Ionizing radiation, Ischemic heart disease, Radiation dose-response relationship, Radiation effects

## Abstract

**Background:**

Studies of cancer survivors treated with older radiotherapy (RT) techniques (pre-1990s) strongly suggest that ionizing radiation to the chest increases the risk of coronary heart disease (CHD). Our goal was to evaluate the impact of more modern cardiac shielding techniques of RT on the magnitude and timing of CHD risk by studying a cohort exposed to similar levels of cardiac irradiation years ago.

**Methods:**

Between 2004 and 2008, we re-established a population-based, longitudinal cohort of 2657 subjects exposed to irradiation for an enlarged thymus during infancy between 1926 and 1957 and 4388 of their non-irradiated siblings. CHD events were assessed using a mailed survey and from causes of death listed in the National Death Index. We used Poisson regression methods to compare incidence rates by irradiation status and cardiac radiation dose. Results were adjusted for the CHD risk factors of attained-age, sex, diabetes, dyslipidemia hypertension and smoking.

**Results:**

Median age at time of follow-up was 57.5 years (range 41.2–88.8 yrs) for irradiated and non-irradiated siblings. The mean estimated cardiac dose amongst the irradiated was 1.45 Gray (range 0.17–20.20 Gy), with 91% receiving < 3.00 Gy. During a combined 339,924 person-years of follow-up, 213 myocardial infarctions (MI) and 350 CHD events (MI, bypass surgery and angioplasty) occurred. After adjustment for attained age, gender, and other CHD risk factors, the rate ratio for MI incidence in the irradiated group was 0.98 (95%CI, 0.74–1.30), and for any CHD event was 1.07 (95%CI, 0.86–1.32). Higher radiation doses were not associated with more MIs or CHD events in this dose range, in either the crude or the adjusted analyses.

**Conclusions:**

Radiation to the heart during childhood of < 3 Gy, the exposure in most of our cohort, does not increase the lifelong risk of CHD. Reducing cardiac radiation to this amount without increasing other cardiotoxic therapies may eliminate the increased CHD risk associated with radiotherapy for childhood cancer. By extension there is unlikely to be increased CHD risk from relatively higher dose imaging techniques, such as CT, because such techniques use much smaller radiation doses than received by our cohort.

## Introduction

Since the late 1980s several studies of cancer survivors treated with older chest radiotherapy techniques have demonstrated that they are at increased risk of cardiac mortality and morbidity [1–8]. Childhood cancer survivors who received mediastinal radiotherapy are particularly at high risk for coronary heart disease (CHD) [9–11], and as a result, radiotherapy techniques have been modified to reduce the dose and volume of the heart exposed to irradiation (e.g. shielding the heart, limiting daily fraction size, lower total doses). However such modifications have been relatively recent so the impact on cardiovascular health during adulthood remains uncertain. Although lower-dose volumes of exposure are thought to not increase risk, studies of populations exposed to lower doses of whole body irradiation such as the atomic bomb survivors have reported an increased risk of cardiovascular morbidity and mortality, but not necessarily from CHD specifically [12–15], with the exception of one historical cohort of individuals treated at the University of Chicago for peptic ulcers [16].

In the first half of the previous century, a misconception of the normal size range of thymus glands in infants and the mistaken belief that an enlarged thymus could lead to status lymphaticus and suffocation [17, 18] led to thousands of infants and children being irradiated for thymic enlargement. In 1951, Louis Hempelmann began a longitudinal cohort to study cancer incidence among patients treated for this condition in Rochester, NY, between 1926 and 1957 and their untreated siblings [19, 20]. The re-initiation of this dormant cohort allowed us to estimate the effect of more modern techniques of chest radiotherapy for childhood and adolescent cancer on coronary heart disease incidence and mortality over most of the lifespan. This is because although this cohort was treated with older techniques, they received total cardiac doses similar to the cumulative doses many children with cancer receive with more modern RT techniques. Additionally since the cardiac doses this cohort received is twenty to 500 times greater than that received for a chest computed tomography (CT), this study may inform the higher end of lifetime cardiac risk of children who receive multiple CT scans [21, 22].

## Methods

### The population-based Hempelmann cohort

This population based cohort and survey methods are described in more detail in elsewhere [23, 24]. In brief, the cohort was formed in the early and mid 1950s by collecting records from all Rochester, New York, area hospitals and clinics, that administered thymic irradiation, except for one practice that closed in 1944 and whose records were destroyed. (This clinic treated fewer than 400 children.) All exposed subjects received orthovoltage irradiation. The number of fractions ranged from one to seven, although 89% of patients received only one or two treatments. Time between the first and last treatment was 90 days or less for 98% of patients; 96% were treated at 1 year of age or less. Non-irradiated siblings born before the third follow-up survey in 1963 were included in the cohort. Subjects were excluded if follow-up ended within 5 years after birth, either from death or loss to follow-up [20]. The studied cohort included 2657 thymic irradiated and 4833 non-thymic irradiated siblings, referred to as the irradiated and non-irradiated siblings, respectively, or subjects collectively, throughout the rest of the paper. Irradiated individuals had a variable number of siblings, including none at all, so direct matching comparisons could not be easily performed.

The cohort was surveyed by mail or telephone 6 times, between 1953 and 1987 [19, 20, 25–28], but these surveys did not collect information related to cardiovascular disease, except whether the respondent smoked. Survey response rates were high and similar among irradiated and non-irradiated siblings. In the 1985 survey, approximately 85% of both groups responded; 5% had died, and 10% declined to participate or were lost to follow-up.

### Data collection

#### Cardiac dosimetry

In the early 1990s, Dr. Stovall and colleagues re-estimated the radiation doses to various organs of each subject, and for the first time estimates were calculated for the heart. The method of dose estimation is described in an earlier publication [24], and used data abstracted from the original patient records including cumulative air dose to the thymus, age at each treatment, treatment field size, thickness of lead protection, kilovoltage, and position of treatment (posterior, anterior, or both). The dose reconstruction methods used are similar to those used in other similar cohorts [29, 30]. Only 4.7% had insufficient data to estimate cardiac dose and were classified as “cardiac dose unknown.” Three subjects had received other radiation treatments concurrently with thymic irradiation. Their doses for this analysis are based solely on their thymic irradiation.

#### Recent follow-up procedures

We re-initiated follow-up of this cohort in 2003 as described previously [24]. Briefly, cohort members were eligible for follow-up if they had returned any of the earlier surveys. During the first year of updating contact information prior to sending out any surveys, we determined that about 11% of the cohort had died and another 10% were not locatable.

Between 2004 and 2008, we collected self-reported data using an 81-item survey. The survey collected information on outcomes and risk factors for cardiovascular disease and cancer. Up to three mailing attempts and four telephone calls were made to each subject.

Data on CHD risk factors were collected primarily in the 2004–2008 survey. However, the 1985 survey also assessed smoking status and hypothyroidism. Subjects who reported having had a myocardial infarction (MI), coronary artery intervention or angina were sent a medical release form so we could obtain relevant medical records. Records were reviewed by 2 of 4 preventive cardiologists/physicians (TAP, RGS, RB, MJA) on a blinded, independent basis in order to determine if a subject had one or more coronary heart disease event(s) reported by the patient or next of kin. Reviewers used the Heart Outcomes Prevention Evaluation Group diagnostic criteria for MI [31]. Along with MI, documented angioplasty and coronary artery bypass were considered to be CHD events, but angiography without treatment and angina without intervention or MI were not. Discrepancies in reviewers’ assessments were resolved by consensus between the two physicians who reviewed a subject’s record.

CHD outcomes were also assessed with cause of death information from the National Death Index (NDI) on the years between its beginning in 1979 and 2005. MI was defined as ICD9 codes 410–12 and ICD10 I21–24. CHD was defined by the following codes ICD9 codes 410–414, 427.5 and ICD10 codes I21-I25, I46. Up to the first 5 causes of death listed were analyzed. We used NDI cause of death data to confirm events reported by next of kin in the current survey, but we did not seek further confirmation of events reported by the NDI alone because of the difficulty in obtaining medical records for these events.

### Statistical methods

We hypothesized that after adjusting for known risk factors, low-dose therapeutic chest radiation would increase the life-long cumulative incidence of MI and CHD, as compared to non-irradiated siblings. We also determined the excess relative risk and excess absolute risk of MI and CHD per Gray (Gy) after adjusting for other CHD risk factors in our sample.

To calculate person-years at risk, we used date of birth as the beginning date for both irradiated and non-irradiated siblings, because 95% of the exposed had been irradiated by 8 months of age. Thus, length of follow-up is nearly equivalent to age at follow-up. The event date was the date of CHD event; data were censored at the most recent survey response. Date of death was only used as an end date if that was the only date we had for an incident CHD event, if that was the last follow-up information we had on the subject (censoring date), or if we received a survey subsequently from next of kin (censoring date or used date of CVD event provided by next of kin).

Incidence rates and their 95% confidence intervals (95% CI) were calculated by irradiation status and dose groups, from which rate ratios adjusted for sex and attained age and their 95% CI were calculated [32]. Potential CHD risk factors and demographic variables (attained age, sex, ever smoked, history of diabetes mellitus, history of dyslipidemia, history of hypertension, and family history of MI and/or sudden death) were compared by thymic irradiation status using Student’s *t*-test for continuous variables and Pearson’s chi-square test for categorical variables. Dyslipidemia was defined as self-reported high cholesterol, high LDL cholesterol and/or high triglycerides on the 2004–8 survey. Data conformed to the assumptions of the tests used to analyze them. These analyses were performed using SAS version 9.2. Multivariate analysis included only subjects who responded to all CVD risk and events questions on 2004–8 survey, i.e. no data imputation methods were used for missing data.

For categorical dose and excess relative risk modeling, we performed multivariate Poisson regression using the AMFIT module in the Epicure statistical program [33, 34]. All statistical tests addressing our main hypotheses were two-sided with an alpha level of 0.05. Person-years were calculated from birth as described above and cross-classified by calendar year, a time-dependent variable of attained age, sex, heart radiation dose, and potentially significant CHD risk factors in our cohort. Model fit was evaluated using two-sided likelihood ratio tests at the 5% significance level [35]. Likelihood-based 95% confidence limits were calculated when possible. Reported rate ratios for the entire cohort were adjusted for those factors that were significant in our most parsimonious models for MI and all CHD events respectively.

Excess relative risk was modeled with respect to the cardiac radiation dose. A typical excess relative risk model used to evaluate linear-dose and dose-squared components was:

$$ \mathrm{ERR}=\uplambda \mathrm{s}\ \exp\ \left(\sum {\alpha}_j{x}_j\right)\ \left(1+{b}_1D+{b}_2{D}^2\right) $$

Where ERR is the excess relative risk of MI/CHD, λs is model stratum baseline rates of MI (or CHD) events (strata by sex and attained age) based on the rates in the non-irradiated siblings, the exponential term x_i_ represents potentially significant independent risk factors for MI (or CHD events and α_i_ represents their individual coefficient estimates. D represents the estimated cumulative heart radiation dose in Gy, and β_1_ and β_2_ represent the coefficientsof effect size for the dose terms.

## Results

### MI/CHD incidence

A total of 3071 subjects, 1303 irradiated and 1768 non-irradiated siblings responded to the current survey, for an overall response rate of 46%, after excluding those known to have died (Table 1). Of these responders, 990 irradiated and 1368 non-irradiated siblings had complete information on CHD risk factors and CHD outcomes. Median age at follow-up of was 57.5 years (range, 47.5–78.3 years) in the irradiated and 57.5 years (range, 41.2–88.8 years) in the non-irradiated siblings. Median estimated cumulative heart exposure was 1.41 Gy (range, 0.17–20.2 Gy; mean 1.45 Gy) among all irradiated individuals in the cohort. The frequency of CVD risk factors differed between sibling groups only for sex and hypothyroidism (Table 2), although each of the CVD risk factors, except hypothyroidism, were significantly associated with having an MI (Table 3).

**Table 1:** Response Rates to the 2004–2008 Follow-up Survey of The Hempelmann Cohort by Thymic Irradiation Status

Response Status	Total, n (%) (*N* = 7490)^a^	Irradiated, n (%) (*N* = 2657)^a^	Non-Irradiated Siblings, n (%) (*N* = 4833)^a^
Deceased	857 (11.4)^b^	345 (13.0) ^b^	512 (10.6) ^b^
Not known to be deceased	6633 (88.6)	2312 (87.0)	4321 (89.4)
Total Surveys Mailed	6633 (100)	2312 (100)	4321 (100)
Responded (completed)^c^	3071 (46.3)	1303 (56.4)	1768 (40.9)
Declined^c^	456 (6.9)	107 (4.6)	349 (8.1)
Did Not Respond^c^	2457 (37.0)	696 (30.1)	1761 (40.7)
Undeliverable^c,d^	648 (9.8)	204 (8.8)	444 (10.2)

^a^ From original study population, 199 irradiated and 220 non-irradiated siblings were excluded from the current cohort because they died before age 5 or were untraceable within 5 years after birth

^b^ As a percentage of the entire cohort

^c^ As a percentage of the cohort not known to be deceased. Columns may not sum to 100% as a result of rounding. The “did not respond” category may contain lost to follow-up individuals, because we included people who did not respond and whose survey packets were not returned by post office

^d^ Contact information could not be confirmed, or survey returned by post office but the individual was not known to be deceased

**Table 2:** Frequency of Cardiac Risk Factors by Thymic Irradiation Status

Risk Factor	Irradiated %	Non-Irradiated %	*P*-value
Female	42.1	49.3	< 0.001
Attained Age (mean)^a^	57.5	57.5	0.99
Ever Smoked^b^	60.6	59.9	0.58
Diabetes^c^	11.1	10.3	0.49
Dyslipidemia^c^	54.9	51.8	0.10
Family history of MI or sudden death^c^	21.0	23.5	0.14
Hypertension^c^	40.7	39.7	0.57
Hypothyroid^b^	5.1	3.5	0.003

^a^At time of last follow-up

^b^Information on this variable collected in prior surveys as well as 2004–08 survey

^c^Data on this variable collected only in the 2004–8 Survey

*MI* myocardial infarction

**Table 3:** Coronary Heart Disease Factors by Thymic Irradiation Group

Variable	% Irradiated (n)	% Non-Irradiated	P-value (Ir vs. Non-ir)	Bivariate Rate Ratio MI (95%CI)^a^	P-value^b^	Adjusted Rate Ratio for MI (95%CI)^c^	Bivariate Rate Ratio CHD (95%CI)^a^	P-value^b^	Adjusted Rate Ratio for CHD (95%CI)^c^
Thymic Irradiation			**< 0.001**		> 0.50			0.29	
Yes	100	–		1.08 (0.82–1.42)		0.98 (0.74–1.30)	1.17 (0.94–1.39)		1.07 (0.86–1.32)
No	–	100		Ref			Ref		
**Sex**			**< 0.001**		< 0.001			< 0.001	
Male	57.9	50.7		**2.75 (2.03–3.78)**		**1.97 (1.44–2.72)**	**2.68 (2.13–3.41)**		**1.88 (1.48–2.40**
Female	42.1	49.3		Ref			Ref		
Attained Age^d^ (years, mean)	57.5	57.5	1.00	**1.11 (1.10–1.12)** Per decade	< 0.001		**1.12 (1.11–1.13)**	< 0.001	**1.10 (1.09–1.11)**
Smoke^e^			0.58		< 0.001			< 0.001	
Ever	52.6 (1397)	51.8 (2504)		**1.79 (1.32–2.48)**		**1.49 (1.09–2.07)**	**1.77 (1.39–2.28)**		**1.41 (1.10–1.82)**
Unknown	13.2 (350)	13.5 (651)							
Never	34.2 (909)	34.7 (1678)		Ref			Ref		
Diabetes^f^			0.49		< 0.001			< 0.001	
Yes	11.1 (145)	10.2 (181)		**3.43 (2.39–4.84)**		**1.80 (1.24–2.59)**	**3.27 (2.44–4.32)**		**1.80 (1.34–2.39)**
Unknown	0.4 (5)	0.2 (4)							
No	88.6 (1154)	89.5 (1583)		Ref			Ref		
Dyslipidemia^f^			0.10		< 0.001			< 0.001	
Yes	54.6 (712)	51.7 (914)		**6.42 (3.99–11.04)**		**4.04 (2.48–7.01)**	**6.16 (4.23–9.34)**		**4.01 (2.78–6.24)**
Unknown	0.4 (5)	0.3 (5)							
No	45.0 (586)	48.0 (849)		Ref			Ref		
Hypertension^f^			0.57		< 0.001			< 0.001	
Yes	40.4 (527)	39.4 (698)		**3.96 (2.76–5.80)**		**2.11 (1.45–3.13)**	**4.06 (3.04–5.50)**		**2.29 (1.70–3.13)**
Unknown	0.5 (6)	0.6 (10)							
No	59.1 (770)	60.0 (1060)		Ref			Ref		
Family history MI or sudden death^f^			0.14		0.001	NS		< 0.001	NS
Yes	21.9 (285)	24.2 (429)		**1.78 (1.25–2.52)**			**1.66 (1.25–2.19)**		
Unknown	19.0 (248)	17.6 (311)							
No	59.1 (770)	58.1 (1027)		Ref			Ref		
Hypothyroid^e^			**0.003**		0.50	–		< 0.001	NS
Yes	4.3 (114)	3.0 (146)		1.06 (0.52–1.89)			1.18 (0.71–1.85)		
Unknown	15.3 (407)	14.3 (689)							
No	80.3 (2135)	82.7 (3998)		Ref			Ref		

Bolded *p*-values are for univariate comparisons that are potentially significant and thus tested in building multivariate models

^a^ Relative risk and 95% CI in this column calculated separately for each variable in Poisson regression model stratified by attained age and sex. Sex evaluated in a model adjusted by attained age only

^b^p-value for likelihood ratio test for model fit compared to model with attained age and sex only

^c^Adjusted rate ratios for variables from most parsimonious multivariate Poisson regression model stratified by attained age and sex

^d^At time of last follow-up

^e^Variable information collected on past surveys as well as 2004–08 survey

^f^Variables information only collected in 2004–2008 survey so numbers are for respondents to most recent survey only

**--** Not significant in univariate analysis by treatment group or outcome status so placement in multivariate model not attempted

NS Potentially significant in univariate analysis stratified by attained age and sex (when appropriate), but does not add significantly to model as determined by Likelihood ratio test versus most parsimonious model

Myocardial infarction occurred in 83 irradiated individuals suffered an MI and 130 non-irradiated siblings over 126,513 and 213,411 person-years respectively (Table 4). The resulting crude rate ratio for MI after thymic irradiation was 1.08 (95%CI, 0.82–1.42). Adjusted for attained age and sex, the rate ratio between exposed and unexposed siblings became 1.02 (95%CI, 0.77–1.34) (Table 4). After adjusting for the other collected CHD risk factors the adjusted rate ratio fell further to 0.98 (95%CI, 0.74–1.30) (Table 4). The 7th column in Table 3 illustrates the rate ratios for all the different variables in this fully adjusted model of CHD incidence.

**Table 4:** Incidence Rates of MI events by Estimated Cardiac Radiation Dose from Hempelmann’s Thymic Irradiation Cohort

Dose (Gy)	Number of Persons	Person Years at Risk	Mean Dose Gy (Std Dev)	Median Dose (Gy)	MI Cases	MI Rate (per 10,000 p-yrs)	Rate Ratio of MI Compared to Non-irradiated (95%CI)^a^	Fully Adjusted Rate Ratio of MI (95%CI)^b^
Non-Irradiated	4833	213,411	–	–	130	6.09	Ref	Ref
Total Irradiated	2657	126,513	1.45 (1.28)	1.40	83	6.56	1.02 (0.77–1.34)	0.98 (0.74–1.30)
0.01–0.99	1058	45,694	0.55 (0.21)	0.25	17	3.72	1.00 (0.58–1.62)	1.34 (0.77–2.20)
1.00–1.99	923	43,610	1.59 (0.15)	1.56	33	7.57	1.31 (0.88–1.90)	1.14 (0.76–1.65)
2.00–2.99	323	17,791	2.45 (0.10)	2.46	20	11.24	1.12 (0.68–1.74)	0.83 (0.49–1.33)
3.00–20.99	229	12,568	4.28 (0.58)	4.00	8	6.37	0.56 (0.56–1.06)	0.74 (0.33–1.42)
Irradiated but dose unknown	124	6850	–	–	5	7.30	0.73 (0.26–1.61)	0.63 (0.22–1.39)
							Test of trend^c^ *P* = 0.10	Test of trend^d^ P > 0.50

Analysis was performed with the AMFIT module of Epicure on all 7490 subjects in the thymus irradiation group

^a^ Rate ratio is based on dose category model adjusted by attained age and sex

^b^ Fully adjusted rate ratio is based on a dose category model adjusted for attained age and sex, diabetes, dyslipidemia, ever smoked, and hypertension

^c^ Test for trend of rate ratios by dose categories. Excludes subjects in the unknown radiation dose category

^d^ Test for trend of rate ratios by dose categories adjusted for attained age, sex, diabetes, dyslipidemia, ever smoking, hypertension

Among irradiated individuals, 144 had a CHD event (MI, coronary artery intervention or angina) as did 206 non-irradiated siblings over 126,244 and 213,024 person-years respectively (Table 5). (Person-years of follow-up vary slightly, because the first CHD event date may differ first MI date.) The resulting crude rate ratio for a CHD event after thymic irradiation was 1.17 (0.94–1.39). Adjusting for attained age and sex gave a rate ratio of 1.12 (95%CI, 0.90–1.39), and 1.07 (95%CI, 0.86–1.32) after adjusting for the other collected CHD risk factors as well (Table 5). The last column in Table 3 illustrates the rate ratios for all the different variables in this fully adjusted model of CHD incidence.

**Table 5:** Incidence Rates of CHD events by Estimated Cardiac Radiation Dose from Hempelmann’s Thymic Irradiation Cohort

Dose (Gy)	Number of Persons	Person Years at Risk	Mean Dose Gy (Std Dev)	Median Dose (Gy)	MI Cases	MI Rate (per 10,000 p-yrs)	Rate Ratio of CHD Compared to Non-irradiated (95%CI)^a^	Fully Adjusted Rate Ratio of CHD (95%CI)^b^
Non-Irradiated	4833	213,024	–	–	206	9.67	Ref	Ref
Total Irradiated	2657	126,244	1.45 (1.28)	1.40	144	11.41	1.12 (0.90–1.39)	1.07 (0.86–1.32)
0.01–0.99	1058	45,659	0.55 (0.21)	0.25	22	4.82	0.81 (0.51–1.24)	1.14 (0.70–1.75)
1.00–1.99	923	43,533	1.59 (0.15)	1.56	51	11.72	1.28 (0.93–1.73)	1.26 (0.91–1.70)
2.00–2.99	323	17,735	2.45 (0.10)	2.46	33	18.61	1.17 (0.79–1.66)	0.96 (0.65–1.37)
3.00–20.99	229	12,486	4.28 (0.58)	4.00	29	23.23	1.31 (0.87–1.89)	1.10 (0.47–1.61)
Irradiated but dose unknown	124	6831	–	–	9	13.18	0.82 (0.39–1.51)	0.65 (0.31–1.20)
							Test of trend^c^ *P* = 0.07	Test of trend^d^ *P* > 0.50

Analysis was performed with the AMFIT module of Epicure on all 7490 subjects in the thymus irradiation group

^a^ Rate ratio is based on dose category model adjusted by attained age and sex

^b^ Fully adjusted rate ratio is based on a dose category model adjusted for attained age and sex, diabetes, dyslipidemia, ever smoked, and hypertension

^c^ Test for trend of rate ratios by dose categories. Excludes subjects in the unknown radiation dose category

^d^ Test for trend of rate ratios by dose categories adjusted for attained age, sex, diabetes, dyslipidemia, ever smoking, hypertension

### Dose effect modeling

**Fig. 1:**
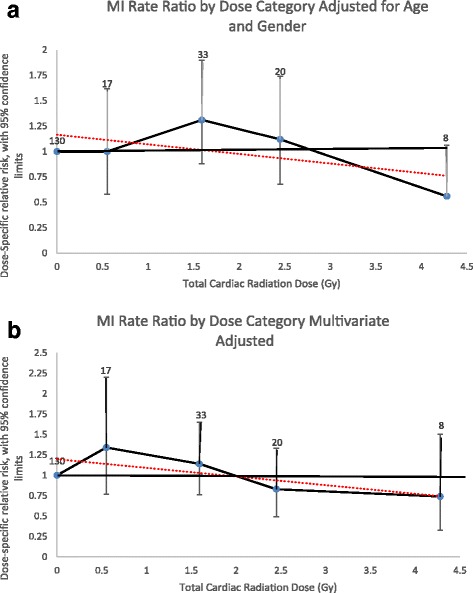
Myocardial Infarction Rate Ratios by Radiation Dose Category. **a** Adjusted for attained age and sex only. **b** Multivariate adjusted (attained age, sex, diabetes, dyslipidemia, ever smoked, and hypertension). Rate ratios plotted by mean dose of intervals evaluated. Number equals number of events for individuals exposed to that dose category. Dotted line equals linear regression line the slope of which is the ERR/Gy estimate

Modeling ERR as a linear function over the entire follow-up period resulted in an excess relative risk per Gray (ERR/Gy) for MI of − 0.05 (95%CI, − 0.13 – 0.08) or − 5% (95%CI, − 13 – 8%) after excluding the individuals with an unknown cardiac radiation dose and adjusting for attained age and sex (Fig. 1a). Adjusting for other CHD risk factors, the ERR/Gy was − 6% (95%CI, − 16 – 6%) (Fig. 1b). The linear dose model did not fit the data better than a model without dose (likelihood ratio test, *p* = 0.42), nor did the linear-quadratic (*p* = 0.27) or quadratic models (*p* = 0.09) fit better than a linear dose model.

**Fig. 2:**
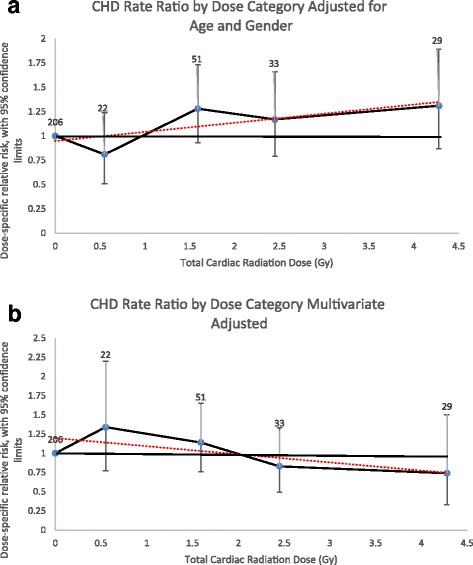
Coronary Artery Disease Event Rate Ratios by Radiation Dose Category. **a** Adjusted for attained age and sex only. **b** Multivariate adjusted (attained age, sex, diabetes, dyslipidemia, ever smoked, and hypertension). Rate ratios plotted by mean dose of intervals evaluated. Number equals number of events for individuals exposed to that dose category. Dotted line equals linear regression line the slope of which is the ERR/Gy estimate. Coronary artery disease events include myocardial infarction, coronary artery bypass surgery and angioplasty

Estimates obtained for the combined CHD events incidence analyses differed. The ERR/Gy for CHD was 8% (95%CI, − 1 – 20%) in a linear dose model adjusted for attained age and sex (Fig. 2a). This linear dose model did not fit the data better than a model without dose (likelihood ratio test, *p* = 0.08). The linear-quadratic (*p* = 0.11) and quadratic models (*p* > 0.50) also did not fit the data better than the linear dose model. The linear model adjusted for all significant CHD risk factors revealed an ERR/Gy of − 3% (95%CI, − 7 – 10%) (Fig. 2b).

## Discussion

Although therapeutic doses of irradiation that includes the heart in the treatment field increase the lifelong risk of cardiovascular disease, and particularly coronary heart disease [1–8, 36, 37], our results indicate that chest irradiation in the range we studied, mostly 0 to3 Gy during early childhood, is unlikely to markedly increase long-term CHD risk in the individual. This dose range is two to three magnitudes greater than that of a chest CT scan, which is typically 7 mGy [21, 22, 38] but one order of magnitude lower than the cumulative doses given for Hodgkin Disease (25 – 40Gy).

Although we found no significant linear dose response, which appears to contradict with the findings of other investigators, our point estimate for ERR/Gy for the incidence of CHD of 8% (95%CI, − 1 – 20%) is actually consistent with what others have found. This is true whether we compare our estimates to those from cohorts exposed to moderate- to low-level, whole body irradiation (atomic bomb survivors and nuclear workers) or to outdated therapies for benign diseases or cancer.

Since we conceived of this study, in 2002, Little et al. published a meta-analysis (8 studies; 635,000 people followed for as long as 53 years) summarizing information on the circulatory disease risks associated with moderate- and low-level, whole-body irradiation (a mean cumulative dose less than 0.5 SV and a daily dose rate less than 10mSV per day) [39]. They estimated an increased relative risk per sievert (ERR/Sv) of 10% (95%CI, 5% – 15%) for ischemic heart disease (IHD), using a fixed effects, no-threshold, linear dose model.

Among the studies included in the meta-analysis by Little et al., two studies evaluated atomic bomb survivors. The first evaluated a smaller group of about 20,000 serially followed survivors (the Adult Health Study population) for cumulative incidence of CHD based on follow-up until 1998. It revealed an excess relative risk for IHD incidence of 4% (95%CI, − 6 – 14%) per Sievert [40]. The estimate for MI specifically was 11% (95%CI, − 10 – 46%), which overlaps with our estimate. Interestingly in survivors less than 40 years old at the time of the bombing, radiation dose was significantly associated with MI incidence, with an ERR/Sv of 25% (95%CI, 0–69%). However, this result was for a quadratic dose relationship. This finding caused us to test quadratic and linear-quadratic dose models as well as linear dose models.

In the second study, published in 2010, Shimizu et al. [13] evaluated circulatory disease mortality up through the end of 2003 in 86,611 Atomic bomb survivors in the Life Span Study performed. They found that a linear model fit overall heart disease best with an ERR/Sv of 14% (95%CI, 6–23%) when calculated over the entire dose range of exposure (< 0.005–2.00+ Sv). However the relationship was no longer statistically significant when the analysis only included survivors with doses less than 0.5 Gy. For IHD specifically, the ERR/Gy was 2% (95%CI,-10–15%), a finding not entirely consistent with a linear dose effect model nor statistically significant. The inability of this analysis to demonstrate significant ERR/Gy for IHD, emphasizes that very large studies with long follow-up periods are sufficiently powered to detect a statistically significant effect of lower doses of irradiation to the heart.

A study quite similar to ours evaluated CHD incidence in patients treated for peptic ulcer disease with radiotherapy or other means between 1936 and 1967 at the University of Chicago. Using dose estimates calculated by the same group that performed our dose estimates (Stovall et al.), Carr et al. [16] reported a dose dependent increase in CHD mortality after an average follow-up of 22.5 years in the 1475 irradiated patients and 27.5 years in the 1568 non-irradiated subjects and after adjusting for several factors including sex and age at treatment. Irradiated patients received average total cardiac doses ranging from 0.1 to 7.6 Gy delivered typically in daily fractions of 1.5 Gy during one or two 6- to 14-day treatment courses. The risk of CHD showed no signs of elevation (RR = 1.00 (95%CI, 0.76–1.33)) in the quarter of patients with cardiac doses less than 2.0 Gy, which is interesting considering that the average cumulative dose of cardiac irradiation in our cohort was only 1.45 Gy. In 2012, Little et al. evaluated the ERR models for CHD mortality in the Carr study and found an ERR/Gy of 10.2% (95%CI, 3.9–17.4%), after adjusting for the two CHD risk factors, smoking and alcohol use, for which they had information [41].

Two more recent studies have evaluated the linearity of CHD mortality with increasing dose in patients irradiated for benign disease. Zablotska et al. evaluated IHD mortality in the Canadian Fluoroscopy Cohort Study that included 63,707 tuberculosis patients exposed to multiple fluoroscopic procedures (mean lung dose, 0.79 Gy; range, 0–11.60 Gy) between 1930 and 1952 and followed for causes of death from 1950 to 1987, for a total of 1.9 million person-years [42]. Although the overall risk of death from non-cancer causes was significantly lower in this cohort than in the general Canadian population (*p* < 0.001), the ERR/Gy of lung irradiation for IHD mortality was 17.6% (95%CI, 1.1–39.3%) after adjusting for dose fractionation. In the smaller Massachusetts tuberculosis fluoroscopy cohort with 13,568 persons, exposed to a mean lung dose of 0.36 Gy (range 0–8.56 Gy) between 1915 and 1968, the linear relationship between lung irradiation and IHD mortality was negative, at an ERR/Gy of − 7.7% (95%CI, − 13 – 1.2%) [43], a similar finding to ours. Not surprisingly, with respect to the studies discussed so far, the Massachusetts cohort is most similar to ours in terms of person-years of follow-up and second most like ours in term of cumulative dose of exposure, second only to the larger Canadian study. Both studies used lung doses which can be slightly lower or up to 2 times lower than the corresponding cardiac dose [44]. Also of interest is, that in the Canadian cohort, time since therapy was associated with a decreasing effect of radiation on IHD mortality, whereas in studies of childhood cancer survivors and in our study time since therapy or attained age independently increased the risk of IHD mortality as discussed below. Quite recently a pooled analysis of these studies was performed [44]. It found that the ERR/Gy for IHD was 27% (95%CI, 0.3–55%) when analyses were restricted to doses < 0.5 Gy but negative − 4% (95%CI -6 – -1%) over the entire range. Indeed for almost all circulatory outcomes, the ERR/Gy estimate was greater the more restricted the dose range analyzed, though the biological basis for such restrictions is not definitive.

Several studies in the last decade have evaluated the ERR/Gy curve for CVD in cancer survivors. Tukenova et al. studied the long-term mortality in 4122 5-year survivors of a childhood cancer diagnosed before 1986 in France and the United Kingdom. Cardiac dose averaged 5 Gy (range 0 - >15Gy) [45]. After an average follow-up of 27 years, the ERR/Gy for all cardiac mortality was 60% (95%CI, 20–250%) after adjusting for sex, age at treatment, duration of follow-up, and other factors. However the increased risk was not statistically significant in the categorical evaluation of dose until exposure was 5.0 Gy or greater. Mulrooney et al. performed similar analyses of CVD incidence in 14,358 five-year survivors and 3899 siblings in the Childhood Cancer Survivors Study surveyed repeatedly between 1994 and 2002 [46]. In addition to increased risk for all CVD outcomes combined, there was a dose-dependent increase in MI incidence, even after adjusting for known CVD risk factors and other cancer therapy factors. The average mean cardiac dose in those irradiated was about 12 Gy (range < 0.1 - > 35.0 Gy). The specific ERR/Gy for the incidence of MI in this cohort was 4.5% (95%CI, 1.0–8.9%) This estimate was not noted in the original paper, but it was reported by Darby et al. in the discussion of their population-based, case-control study of major coronary events (i.e. MI, coronary revascularization, or death from IHD) in 2168 women who underwent radiotherapy for breast cancer between 1958 and 2001 in Sweden and Denmark [37]. The average, whole cardiac dose was 4.9 Gy (range, 0.03–27.72 Gy). Incidence rates of major coronary events increased linearly cardiac dose [ERR/Gy of 7.4% (95%CI, 2.9 to 14.5%)], with no apparent threshold.

Comparing our findings to others is complicated by several reasons. In the low-dose studies reviewed by Little et al. in 2012 [39], the exposures were whole-body exposures and, with the exception of the atomic bomb survivors, were at a very low dose rates over a long period. Thus, the balance of pathological mechanisms causing different forms of CVD is likely to be different than in our population [47–49]. Additionally, some of the studies discussed used effective doses (Sieverts) in their evaluations, whereas we used absorbed doses (Gray) to the heart. Although with gamma-rays and X-rays these units should be equivalent, the difference does make comparisons confusing. Even when absorbed doses were used, doses to different target organs were used for modeling ERR, such as in the TB studies which used lung doses. In comparing our study to those in cancer survivors, our cohort’s average cardiac dose (and dose range) was much lower, though more similar in that treatment was targeted. On the other hand the effect of cardiac irradiation on CHD in cancer survivors is confounded and likely increased by dysregulation of inflammation from cancer and its treatment with other agents.

### Strengths and limitations

The most notable limitation of our study is its dependence upon self-report and national death records to obtain data on CHD incidence. Besides the potential shortcomings of such data in terms of accuracy, this dependence also limited our ability to detect CHD deaths before 1970, the beginning of the NDI, and in non-respondents to the 2004–2008 survey, given that it was the only survey to ask about CHD incidence. The limited ability to detect events in earlier years is mitigated by the fact that CHD incidence increases with age, being fairly uncommon at less than 40 years of age.

Regarding the accuracy of self-reported data, among participants for whom we could obtain medical records, 65% of self- reported MIs that were confirmed to have occurred at the reported time and another 13% had evidence for a probable MI or prior MI. Because we only obtained medical records from a minority of those reporting an MI or CHD event, we did not formally integrate confirmation information into our analyses.

Another issue is that we may have underestimated the absolute incidence rates of events by including person-years during which we could not have detected an event. We included the person-years of non-respondents to the 2004–2008 survey up until their last survey response among the prior surveys, even though these questionnaires did not ask about CHD. Nevertheless we performed analyses that started follow-up from age 15 year because the first self-reported case of CHD occurred at age 16 years, which helps minimize these potential extra person years; the results were remarkably consistent (data not shown). The issues related to event ascertainment and calculating person-years of follow-up should have affected both irradiated and non-irradiated siblings equally leading to non-differential bias. Further, the effect of radiation on heart disease was likely not well known among the general population at the time our survey. Additionally, our survey asked about multiple outcomes without stating that our main interest was CHD; prior questionnaires did not ask about the occurrence of CHD at all.

Another limitation of the study is the fairly low response rate in the 2004–2008 survey which differed between the irradiated and non-irradiated siblings. Given the lower response in non-irradiated siblings, if anything we most likely underestimated the rate of the disease in this comparison group, thus overestimating any effect of irradiation. Yet, we found little effect at the doses to which our cohort was exposed. Additionally, in an earlier article on thyroid cancer using factors collected in the 1985–1987 survey, we evaluated whether this response pattern might lead to differential non-response bias, threatening internal validity [23]. Of the 13 factors compared, the only CHD risk factors that significantly differed between responders and non-responders for only one group were smoking and hypothyroidism. Smoking was underrepresented in the non-irradiated sibling respondents (54.7% vs 61.7%, *p* < 0.001), and hypothyroidism was overrepresented in the irradiated individuals (6.0% vs. 3.9%, *p* = 0.026).

Finally our outcome mixed CHD incidence and mortality. Radiation probably acts through separate yet overlapping mechanisms to increase incidence and mortality, which may differ by exposure levels [48]. The first mechanism increases inflammation in the coronary arteries, enhancing the development of coronary atherosclerosis. The latter mechanism increases inflammation in the cardiac microcirculation damaging the heart and causing any age-related MI to be more lethal even if the frequency of coronary ischemia is the same. Arguably, these mechanisms suggest that lower irradiation exposures could increase the lethality of MI and CHD without increasing the incidence. We studied primarily incidence whereas the studies most similar to ours studied mortality, so the above might help explain why our effect size differs from most previous studies.

Our study has several strengths. First, to our knowledge, the median time since irradiation in the Hempelmann Cohort is longer than that of any other radiation-exposed cohort followed for cardiovascular disease, other than the atomic bomb survivors’ cohort [13] and parts of the Massachusetts tuberculosis cohort [43]. Second, in our study, the sibling comparison group helps control for confounding from family history and potential risk factors that were not collected but are related to upbringing. Third, although the radiation received by our cohort differs from that used today in terms of dose distribution and less-precise techniques, it is more similar to the therapeutic and diagnostic radiation received by patients today than is the whole-body radiation received by atomic bomb survivors or occupationally exposed cohorts. Fourth, our cohort was exposed during childhood and not adulthood, as were most of the exposures in the tuberculosis cohorts and the entire peptic ulcer disease cohort. Finally, radiation was not administered in response to cancer, so our findings are not confounded by the possibility that an initial malignancy or other therapies increased the risk of coronary heart disease.

## Conclusions

We did not find a significant effect of therapeutic chest irradiation during childhood in the dose range studied (cardiac dose, mostly 0 - 3Gy) on the cumulative incidence of CHD or MI up to an average of 57.5 years after exposure. At worst, with such exposures over the life-span, the risk of a CHD event is increased by 7%, although this increase is neither statistically significant nor clinically important compared to the risks imposed by traditional cardiovascular disease risk factors. This finding, and the existing literature on childhood cancer survivors, suggests that limiting cardiac exposures to such low therapeutic doses without increasing other cardiotoxic therapies might eliminate the increased risk of CHD from chest irradiation for childhood cancer treatment. Additionally. because exposures in our cohort were about 200 times higher than that for a chest or abdominal CT in children, our results suggest relatively higher dose imaging techniques such as CT, is unlikely to increase CHD risk.

Nevertheless, our estimated 8% (95% CI, − 1% to 20%) excess relative risk per Gy of cardiac irradiation on the incidence of CHD is consistent with what others have found regarding IHD mortality and incidence, though the latter has been studied less. In other radiation exposed cohorts, whether of atomic bomb survivors, those exposed to radiation for benign diseases, or cancer survivors, estimates of ERR/Gy for IHD have ranged from − 7.7% to 17.6% with the best fit generally being a no-threshold, linear-dose model. Thus although our results did not fit a linear dose model with statistical significance, our findings also do not provide substantial evidence to refute such a model. This is because our estimates are rather consistent with the literature, and the relatively smaller size of our study leads to less precise estimates.

## Abbreviations

CHD: Coronary heart disease
CI: Confidence interval
CT: Computed tomography
CVD: Cardiovascular disease
ERR: Excess relative risk
Gy: Gray (standard unit of absorbed irradiation)
ICD: International Classification of Diseases (ICD-9 or − 10, International Classification of Diseases, Ninth Revision or 10th revision)
IHD: Ischemic heart disease
MI: Myocardial infarction
NDI: National Death Index
Sv: Sievert (standard unit of effective irradiation) For gamma rays and x-rays 1 Sv = 1 Gy)
